# Zinc and/or Selenium Enriched Spirulina as Antioxidants in Growing Rabbit Diets to Alleviate the Deleterious Impacts of Heat Stress during Summer Season

**DOI:** 10.3390/ani11030756

**Published:** 2021-03-10

**Authors:** Fawzia Hassan, Samia Mobarez, Manal Mohamed, Youssef Attia, Aml Mekawy, Khalid Mahrose

**Affiliations:** 1 Department of By-products Utilization Research, Animal Production Research Institute, Agricultural Research Center 12618, El-Dokki, Giza 12611, Egypt; fawzia_amer@yahoo.com; 2 Department of Poultry Nutrition Research, Animal Production Research Institute, Agricultural Research Center 12618, El-Dokki, Giza 12611, Egypt; samiamostafa116@gmail.com (S.M.); manalsaudy@yahoo.com (M.M.); 3 Department of Agriculture, Faculty of Environmental Sciences, King Abdulaziz University, Jeddah 21589, Saudi Arabia; yaattia@kau.edu.sa; 4 Poultry Production Department, Agriculture College, Damietta University, Damietta 34516, Egypt; amlmekawyaa@gmail.com; 5 Animal and Poultry Production Department, Faculty of Technology and Development, Zagazig University, Zagazig 44511, Egypt

**Keywords:** antioxidants, growth, heat stress, rabbit, selenium, spirulina, zinc

## Abstract

**Simple Summary:**

Heat stress in summer season impairs growth and causes heat-induced physiological stress in rabbits. Zinc acts as an antioxidant stress agent by inhibition of oxidation of macromolecules such as DNA as well as inhibition of inflammatory response, eventually resulting in the down-regulation of reactive oxygen species production. Selenium is a powerful biological anti-oxidant mineral. Spirulina is comparatively confined extreme protein (55–65%) and comprised all important amino acids, has wellbeing assistances, immuno-stimulatory influences and antiviral activity and ensured the capability to diminish heat stress impacts. In the current work, effects of dietary supplemental zinc and/ or selenium enriched spirulina (100 mg Zn-Sp/kg diet, 0.5 mg Se-Sp/kg diet or 100 mg Zn-Sp+ 0.5 mg Se-Sp, respectively) as antioxidants on growth performance, nutrient digestibility, plasma biochemicals and antioxidant status of New Zealand White growing rabbits under summer conditions were evaluated. The findings showed that the supplemented diets enhanced growth performance of rabbits at marketing, hot carcass weight, dressing percentage, high density lipoprotein cholesterol and total antioxidant capacity and reduced thio-barbituric acid reactive substances. Finally, dietary supplementation of 100 mg Zn-Sp, 0.5 mg Se-Sp or their combination could improve growth performance, nutrients digestibility and antioxidant status of heat stressed growing rabbits.

**Abstract:**

Effects of dietary supplemental zinc and/ or selenium enriched spirulina (Zn-Sp, Se-Sp and Zn-Se-Sp, respectively) as antioxidants on growth performance, nutrient digestibility, plasma biochemicals and antioxidant status of growing rabbits under summer conditions were evaluated. A total of 160 New Zealand White male rabbits at six-weeks-old were randomly assigned to four groups. The first group received untreated diet (control). The other groups received diet supplemented with 100 mg Zn-Sp/kg diet, 0.5 mg Se-Sp/kg diet or 100 mg Zn-Sp+ 0.5 mg Se-Sp, respectively. The findings showed that the supplemented diets enhanced growth performance of rabbits at marketing. Rabbits fed Zn-Sp exhibited high dry and organic matter digestibilities while those fed Zn-Sp and Zn-Se-Sp diet supplemented achieved high crude protein digestibility. Rabbits fed diet supplemented with Zn-Se-Sp gave the highest hot carcass weight when competed with their counterparts. Zn-Sp and Zn-Se-Sp supplemented diets tended to promote dressing percentage. Low concentrations of plasma total cholesterol, LDL-cholesterol and VLDL-cholesterol were recorded by Se-Sp and Zn-Se-Sp groups. Rabbits fed Se-Sp, Zn-Se-Sp had the greatest HDL, plasma TAC and catalase and the lowest TBARs. Conclusively, dietary supplementation of 100 mg Zn-Sp, 0.5 mg Se-Sp or their combination could improve growth performance, nutrients digestibility and antioxidant status of heat stressed growing rabbits.

## 1. Introduction

The environmental and nutritional factors are affecting the intensive rabbit production [1,2]. Rabbits play an increasingly important role in meat production throughout the world [3]. Growing rabbits are very susceptible to high temperature and the heat anxiety in summer season is correlated with reduces in growth performance and increases in mortality [2,4]. Oxidative stress refers to the imbalance between free radicals production and the ability of the antioxidant defense system of the body to detoxify or impair oxidative damage to DNA, proteins, and lipids [5].

Zinc (Zn) is a component of more than 300 enzymes and more than 2000 transcriptional factors and is involved in the biosynthesis of nucleic acids and in cell division processes [6,7]. Practical commercial rabbit diets include a broad range of zinc levels (40–140 mg/kg). Growing rabbits respond positively to 100 mg zinc/kg diets in terms of improving body weight gain (BWG) and feed conversion ratio (FCR) [8]. Besides, zinc acts as an antioxidant stress agent by inhibition of oxidation of macromolecules such as DNA and proteins as well as inhibition of inflammatory response, eventually resulting in the down-regulation of reactive oxygen species production [5].

Selenium (Se) is a powerful biological antioxidant mineral. It can control several vital biological processes [9,10]. Moreover, Se is an integral component of at least 25 selenopro-teins and serving as an essential co-factor in the antioxidant enzyme system. The intake of Se in productive animals affects nutrient utilization, productive performance, antioxidative mechanism, reproductive function, hormone metabolism and responses of the immune system [11,12,13].

Spirulina platensis have been exhausted for several years as nourishment for people and animals owing to the outstanding nutritious profile and great carotenoid substance. Spirulina is comparatively confined extreme protein (55–65%) and comprised all important amino acids [14,15], has wellbeing assistances [16], immuno-stimulatory influences and antiviral activity [15,17] and ensured the capability to diminish heat stress impacts [18].

The purpose of this study was to investigate the effects of dietary supplementation of Zn- and/ or Se-enriched Spirulina or their combination on growth performance, plasma biochemicals and antioxidant status of growing New Zealand White rabbits (NZW) during summer season (Julie and August).

## 2. Materials and Methods

The current study was performed at Borg-El Arab, Alexandria Governorate, Animal Production Research Institute, Agricultural Research Center, Ministry of Agriculture, Egypt.

*Spirulina platensis* (*Arthrospira platensis*) was obtained from Agricultural Microbiology Department, National Research Centre (NRC), Giza, Egypt. The principles of the cultivation system and modification of culture medium by the addition of inorganic zinc and selenium sources were described [19]. The biomass concentration was 1 g of dry mass/L. Resultant Zn-enriched Spirulina contained 100.17 mg Zn for each 1 gm dry algae. Selenium enriched algae is produced by growing strain of Spirulina platensis, algae containing 1 mg Se/g algae.

A total number of hundred and sixty weaned New Zealand White (NZW) male rabbits, 6-weeks old (average initial live body weight; 744.79 ± 17.56 g), were randomly assigned to four experimental groups (40 rabbits/each; 5 replicates, 4 rabbits/each). Rabbits were housed an open-sided house in individual cages (60 × 40 × 24 cm). Feed and water were offered ad libitum throughout the experimental period (6–14 weeks of age). Environ-mental temperature and RH were noted daily, and then averages of temperature, RH and temperature-humidity index (THI) for two months (Julie and August) were estimated [20] expending the next principle: THI = db °C-{(0.31−0.31RH)(db °C−14)}, where db °C is the dry bulb temperature in Celsius and RH is the relative humidity %; the assessed estimates of THI were categorized [20] as follows: < 22.2 is lack of heat anxiety, 22.2− < 23.2 is reasonable heat anxiety, 23.3− < 25.5 is acute heat anxiety, and 25.5 or more is extremely acute heat anxiety. Four pelleted diets were formulated and nutrients requirements were adjusted [21] as shown in Table 1. The first experimental group received untreated diet (control). The second, third and fourth experimental groups received diet supplemented with 100 mg Zn-Sp/kg diet, 0.5 mg Se-Sp/kg diet, 100 mg Zn-Sp+ 0.5 mg Se-Sp, respectively. Body weight (BW) and feed intake (FI) were recorded weekly and then BWG and FCR were computed.

At the end of the experimental period, digestibility trial was carried out on ten rabbits per group. Rabbits were housed individually in metabolic cages (1825 mm height × 1370 mm length × 840 depth including feeders × 1210 width when the door is opened) which allowed for the collection of feces and urine separately for five consecutive days collection according to European reference method for rabbit digestion trials [22]. The experimental diets were offered daily and fresh water was provided all times. During the collection period, feces were collected every 24 h for 5 consecutive days, daily FI and feces excreted were accurately determined. Feces of each animal were dried, ground and stored until analysis. Digestible energy (DE, Kcal/Kg diet) was calculated as follow: TDN (Total Digestible Nutrients) × 44.3 [23].

Chemical analyses of both experimental diets and feces were [24] for determining moisture, crude protein (CP), crude fiber (CF), ether extract (EE), nitrogen free extract (NFE), ash. Calcium and Zinc were determined by atomic absorption spectrophotometer and phosphorous was determined colorimetrically using spectrophotometer (3300 perken Elmer, California, United States).

At the end of the experimental period, six male rabbits from each group were randomly taken, fasted for 12 h, individually weighed and immediately slaughtered. Slaughter procedure and carcass analysis were carried out [25]. After complete bleeding, pelt, viscera and tail were removed and then the carcass and giblets (liver, heart, and kidney) were weighed. Dressing percentage included relative weights of the carcass, giblets and head were estimated. Blood samples (5 mL from each rabbit) were collected at slaughtering time (during bleeding) in heparinized glass tubes. Blood plasma was separated by centrifugation at 3000 rpm for 15 min. The collected plasma was stored at −20 °C until assay. Plasma total protein, albumin, total cholesterol, LDL, HDL-cholesterol, aspartate aminotransferase (AST) and alanine aminotransferase (ALT) were colorimetrically determined using commercial kits (acquired from Bio-diagnostic, Giza, Egypt), according to the manufacturers’ instructions. Plasma total protein, albumin, cholesterol, LDL-cholesterol, HDL-cholesterol and trans-aminases were determined [26,27,28,29,30,31]. Plasma globulin values were obtained by subtracting albumin values from the corresponding total protein values. The albumin/globulin ratio was calculated. Zinc and selenium concentrations in plasma were determined by using atomic absorption analysis. Plasma total antioxidant capacity (T-AOC) was measured [32]. Thiobarbituric acid reactive substances (TBARS), superoxide dismutase enzyme (SOD), catalase (CAT) and glutathione peroxidase enzyme (GSH-Px) were verified by colorimetric techniques using a commercially obtainable kit (Bio-diagnostic., Cairo, Egypt).

The obtained data were statistically analyzed by one-way analysis of variance using the general linear model procedure of SAS^®^ Software Statistical Analysis [33]. Differences among treatment means were tested by Duncan’s Multiple Range-Test [34].

## 3. Results

Temperature and relative humidity values in this work ranged between 33.74 to 34.82 °C and 83.83 to 84.39%, correspondingly. Estimates of THI extended amid 32.57 to 33.79 through the investigational interval, representing exposure of NZW growing rabbits to extremely acute heat anxiety.

Growth performance evaluation of rabbits during the different experimental periods is presented in Table 2. Groups of rabbits consumed diets supplemented with Zn- and Se-enriched SP or their combination had significantly (*p* < 0.01) greater BW at 14 weeks of age (*p* = 0.005) and BWG during 6–14 weeks of age (*p* = 0.006). Rabbits fed diet supplemented with Se-Sp achieved the greatest (*p* < 0.05) BWG when compared with the control, and without significant variations with the other groups of supplementation, during 6–10 weeks of age. However, rabbits fed diet supplemented with Zn-Se-Sp exhibited better (*p* < 0.05) BWG than those of the control group during 10–14 weeks of age. Average of FI was decreased (*p* < 0.05) in rabbits fed diet supplemented with Se-Sp and Zn-Se-Sp compared to the control group during 10–14 weeks of age. There was insignificant difference in average FI among the tested groups through 6–10 and 6–14 weeks of age. Regarding FCR, the rabbit groups fed the supplemented diets presented inferior (*p* < 0.01 and 0.001) FCR than the control group through 6–14 weeks and 10–14 weeks of age.

Results found in Table 3 showed that rabbits fed Zn-Sp recorded significantly (*p* < 0.05) higher digestibility of DM and OM compared to the control group. Rabbits fed diet supplemented with Zn-Sp and Zn-Se-Sp had higher (*p* < 0.05) CP digestibility than the control group. Besides, EE and NFE digestibilities were greater (*p* < 0.05) in rabbits fed diets supplemented with Zn-Sp and Zn-Se-Sp compared to the control group. There were non-significant variations in DM, OM, CP, EE and NFE between the supplemented diets. On the other hand, the different supplementations had no significant effect on CF digestibility. Data of the nutritive values including DCP, TDN and DE illustrated insignificant alterations were noticed between all groups under investigation (Table 3).

The impacts of Zn, Se-enriched SP and or combination on carcass traits of rabbits are displayed in Table 4. All carcass traits, except for pre-slaughter weight and spleen percentage, were significantly changed (*p* < 0.05, 0.01 and 0.001) due to the tested supplementations. Growing rabbits fed diet supplemented with Zn-Se-Sp had the highest (*p* < 0.05) hot carcass weight when competed with their counterparts. The addition of Zn-Sp and Zn-Se-Sp in diets tended to promote (*p* < 0.01) dressing % when compared with the control. Rabbits fed diet containing Se-Sp and Zn-Se-Sp had lower (*p* < 0.05) liver % than the control group, while rabbits fed diets supplemented with Se-Sp and the control group had higher (*p* < 0.05) heart % as compared to those fed diet containing Zn-Se-Sp. However, rabbits fed diets supplemented with Zn-Sp had higher (*p* < 0.05) kidneys % compared to rabbits fed Zn-Se-Sp diet. Rabbits of the control group had greater (*p* < 0.05) giblets % and non-edible parts than those fed diet containing Zn-Se-SP. The rabbits fed diet supplemented with Zn-Se-Sp had worthier (*p* < 0.05) total edible parts than the control group. Meat of rabbits fed diets supplemented with Se-SP and Zn-Se-Sp had lower (*p* < 0.05) values of EE than those of the control. Regarding Zn and Se content of meat, rabbits consumed diets supplemented with Zn-Sp and Zn-Se-Sp had greater (*p* < 0.001) Zn content of meat than those of the control and those fed Se-Sp diet. Meat of growing rabbits fed Zn-Se-Sp and Se-Sp presented higher (*p* < 0.001) content of Se than the control and those fed Zn-Sp diets (Table 4).

As shown in Table 5, plasma total protein levels were higher (*p* < 0.05) in rabbits fed diet supplemented with Zn-Se-Sp compared to the control group. Meanwhile, the same group and those fed Se-Sp recorded higher (*p* < 0.05) globulin levels compared to the control group. While, no significant effect on albumin, A/G ratio, AST and ALT group were observed among all the tested groups and the control group. Additionally, lower plasma total cholesterol, LDL-cholesterol and VLDL-cholesterol concentrations were observed for rabbits given Se-Sp and Zn-Se- Sp diets in comparison with rabbits given the control diet. In the opposite direction, Se-Sp, Zn-Se-Sp were greater (*p* < 0.05) in HDL than the control group. Plasma lipids and triglycerides levels were significantly decreased (*p* < 0.05) with dietary supplementation of Zn-Sp, Se-Sp and Zn-Se-Sp.

Data in Table 5 postulated a significant (*p* < 0.05) increase in plasma T-AOC and catalase levels in rabbits fed Se-Sp and Zn-Se-Sp compared to the control group. On the other hand, a significant decrease (*p* < 0.05) in TBARs was noticed in rabbits fed diet containing Se-Sp and Zn-Se-Sp compared to the control group and Zn-Sp diet. Rabbits fed diet supplemented with Zn-Se-Sp tended to increase (*p* < 0.05) GSH-Px level compared to the other tested groups. As well, the SOD concentration was found to be significantly higher in the rabbits fed diet included Zn-Se-Sp than in rabbits given Zn-Sp containing diets and those fed the control diet. Moreover, SOD levels were higher (*p* < 0.05) in Se-Sp group than those of the control one.

## 4. Discussion

The present study demonstrated that Zn- or Se-enriched Sp or their combination improved BW at marketing, BWG and FCR of growing rabbits, while, these supplementations decreased average FI. Similar to the present findings, Hassan et al. [35] reported that dietary Zn-Sp supplementation at levels of 50, 75 and 100 mg/kg diet caused an increase in marketing BW and improved BWG and FCR. In this regard, Hassan et al. [36] stated that dietary supplementation of Se-algae at 0.05, 0.1, 0.2, 0.4 and 0.5 mg had a positive impact on growth performance of growing rabbits. On contrary, Hosny et al. [37] found non-significant impact on BW and FI of rabbits fed diet including 0.3 mg organic Se/kg diet.

The present study also revealed that the combination effect of Zn and Se-enriched Spirulina showed better growth performance compared to using each alone, this improvement may be due to greater bio-efficacy of Zn-Sp. It could provide more Zn for absorption and resulted in improved growth performance (Hassan et al. [35]. Besides, zinc acts as an antioxidant stress agent by inhibition of oxidation of macromolecules such as DNA and proteins (Prasad and Bao [5]. As well, a potential for better growth may be the profile of organic compounds of Spirulina in the Se-algae (Larsen et al. [38]. Such enhancement of supplementation may be due to the synergetic effects of organic Zn and Se and they have a potential nutritive value as feed additives for growing rabbits under summer heat stress conditions. Furthermore, confirmation is associated with the use of Spirulina which can improve the growth performance because it has some natural constituents such as phycocyanin, beta-carotene, tocopherols, linolenic acid, minerals, vitamins and phenolic compounds that had been shown to have strong antioxidant properties with promote growth and maintain health (Michalak and Mahrose [15] and Farag et al. [16]. It has very high content of macro and micronutrients, essential amino acids, proteins, lipids, vitamins, minerals and anti-oxidants Soni et al. [39]. Furthermore, it is a strong antioxidant due to the presence of high content of antioxidant phenols or flavonoids Gabr et al. [40].

The dietary Zn-Sp supplementation resulted in an escalation in DM, OM. Zn-Se-Sp improved CP, EE and NFE digestibility compared to the control group. In this direction, Hassan et al. [36] found that rabbits fed diets supplemented with Zn-Sp at 100 mg/kg diet led to a rise in all nutrient digestibilities. Our results suggested that supplemental Zn-Sp or Zn-Se-SP to the growing rabbits improved nutrients digestibility. This improvement may be related to the role of zinc in metabolism, whereas zinc participates actively in protein synthesis, carbohydrate and lipid metabolism Chrastinová et al. [41] as well, organic Zn has been considered as an alternative to inorganic Zn in the diets of rabbits and broilers due to its better absorption and efficiency Hassan et al. [35]. Furthermore, organic Se is metabolized much more efficiently the inorganic Se forms and could be efficiently utilized for synthesis of selenoproteins under stress conditions Qazi et al. [42]. The positive effect of Zn-Se-Sp on the digestibility of nutrients may be revealed that Se improved the antioxidative status of rabbits Hassan et al. [43]. Moreover, Zn has an oxidative activity Prasad and Bao [5] which reduces the oxidative capacity and improve animal health.

The effect of Zn-Se-Sp was positive in hot carcass weight, dressing% and total edible parts compared to the control group. The current results are in line with those of Hassan et al. [36] who stated that rabbits on diets supplied with Se-algae at level of 0.5 mg/kg diet increased hot carcass weight, dressing% and total edible parts%. Moreover, Hassan et al. [35] indicated that Zn-Sp supplementation at 50, 75 and 100 mg/kg diet increased hot carcass weight, dressing and total edible parts% compared with the rabbits fed the control diet. On the contrary, Selim et al. [8] postulated that rabbits fed diets containing 50, 100, 200 or 400 mg zinc oxide /kg diet did not change carcass traits. Moreover, no significant effect of Se-algae addition on carcass yield of rabbits was noticed (Marounek et al. [44].

Regarding meat composition, our results agreed with the findings obtained by Hassan et al. [43] who mentioned that dietary addition of Se-algae in rabbit diets at level of 0.2 mg/kg diet decreased EE content of meat. Similarly, Hassan et al. [36] reported that 0.5 mg Se algae/kg diet decreased EE content of rabbit meat. Marounek et al. [44] stated that supplemental Se in the rabbit diet had no impact on CP content of meat. However, Se content in the hind leg meat was found to be increased to dietary Se-alga at 0.2 mg/kg diet [43]. Comparable findings were reported by Marounek et al. [44] and Amer et al. [45] who found that Se-yeast supplementation in rabbit diet deposited in the meat and improved meat quality.

The present study showed a positive influence of the supplemented diets on plasma biochemistry indices. These outcomes are consistent with those of Hassan et al. [36] who mentioned that rabbit consumed diet including 50, 70 and 100 mg/kg had high total protein and HDL. In addition, the dietary Zn- and Se-enriched Sp or their combination had no significant effect of A/G ratio, albumin levels and activities of AST and ALT. Similarly, Hassan et al. [35] concluded that Zn-Sp supplementation did not impact AST, ALT, albumin and A/G ratio. Hassan et al. [43] revealed an increase in plasma total protein concentration as a result of Se-algae addition at level of 0.2 mg/kg diet. Likewise, El-Kholy et al. [46] stated that the addition of different forms of Se led to increases in total protein and globulin levels and did not change ALT and AST activities. The reduction in plasma total cholesterol, LDL-cholesterol and VLDL-cholesterol levels in rabbits fed the supplemented diets may be due to that Zn inhibits the lipolysis in adipose tissues, reduces free fatty acid release into the circulation and its availability to the liver and excessive lipoprotein synthesis (Dieck et al. [47]. Besides Zinc contribution to insulin secretion and action, Zinc directly affects lipid metabolism then increased free fatty acid flux to the liver which stimulates the assembly and secretion of vLDL resulting in hypertriglyceridemia (Ranasinghe et al. [48]. On the other hand, our findings matched with El-Kholy et al. [46] who observed that rabbits received either organic or inorganic Se forms had lower total cholesterol and LDL levels than those of the control group. Moreover, Hassan et al. [35] showed that supplemental Zn-Sp at 75 mg/kg diet decreased in serum total cholesterol and LDL concentration.

It is noteworthy that the supplemental Zn-Se-Sp had a potential antioxidant effect on the rabbits under high temperature and was associated with the lower of TBARs, the higher TAC, GSH, SOD and catalase activities. So it may protect the tissues against oxidative damage which included protein and fat oxidation of growing rabbits under hot conditions. The present results are in agreement with the findings of Alissa et al. [49] who showed that plasma TBARs concentration was reduced by zinc supplementation (0.5%, *w/w*) in rabbit diets and suggested that zinc was associated with a reduction in plasma lipid peroxides. Similar findings have been also reported by Zhang et al. [50] who indicated that rabbits fed a diet containing 0.24 mg/kg Se had the greatest serum GSH-Px and CAT activities. Moreover, there was an increase in the serum T-AOC concentration due to Zn-Sp supplementation at levels of 50, 75 and 100 mg/kg diet [35]. The addition of organic Se at 0.3 mg/diet increased glutathione peroxidase activity in rabbits [37]. The findings reported by Prasad and Bao [5] strongly suggested that zinc reduces oxidative stress and ROS-mediated inflammatory responses, and that zinc acts as a potent agent by inhibition of ROS production and inflammation. Whereas, Zn has a potential role as an antioxidative stress agent, and a pro-antioxidant effect or protective effect against oxidative stress in biological system [5]. In addition, Se positivity affects the antioxidative status of rabbits; this effect has been attributed to selenium which is an essential constituent of GSH-Px [9,10,12]. Glutathione peroxidase helps in protecting cellular membranes from oxidative damage which resulted in enhancing the growth performance of rabbits under hot conditions [51]. Recent literature have shown that Spirulina has an antioxidant, immunomodulatory, anti-inflammatory, antiviral, and antimicrobial activity in various experimental animals [15,40,52]. In this respect, Park et al. [52] suggested that dietary Spirulina supplementation at levels of 0.25, 0.5, 0.75, or 1.0% in broiler diets caused an increase in the serum SOD, and GSH-Px activities. As observed in this study, supplementing rabbit diets with Zn- and Se-enriched SP enhanced the antioxidative status as they are an efficient scavenger of free radicals [53,54].

## 5. Conclusions

It is clear from the present study, that supplementation of 100 mg Zn-Sp, 0.5 gm Se-Sp and or their combination could improve growth performance, nutrients digestibility and antioxidant status of heat stressed growing rabbits.

## Figures and Tables

**Table 1:** Feed ingredients and chemical composition of rabbit basal diet (%DM basis).

Feed Ingredient	(%)	Nutrient Composition (%DM Basis)	
Soybeanmeal (44%CP)	19.30	Dry matter (DM)	88.90
Barley	17.10	Organic matter (OM)	90.70
Wheat bran	24.88	Crude protein (CP)	17.57
Yellow corn	7.00	Crude fiber (CF)	13.01
Clover hay	25.00	Ether extract (EE)	2.01
Molasses	3.00	Nitrogen free extract (NFE)	58.11
Limestone	1.08	Ash	9.30
Di-calcium phosphate	1.71	NDF	29.85
DL-Methionine	0.28	ADF	17.02
Sodium chloride	0.35	ADL	3.44
Vit.-Min. premix ^a^	0.30	Methionine ^b^	0.68
Total	100	Lysine ^c^	0.99
		Calcium	1.27
		Available Phosphours	0.55
		Digestible energy (Kcal/Kg DM) ^d^	2599.49
		Zn (mg/kg DM) ^e^	107.81
		Se (mg/kg dM) ^f^	0.82

^a^ Vit. And Min. premix per kg contains: Vit A 6000 IU; Vit D3450 IU; Vit E 40 mg; Vit K3 1 mg; Vit B1 1 mg; Vit B2 3 mg; Niacin 180 mg; Vit B6 39 mg; Vit B12 2.5 mg; Pantothenic acid 10 mg; biotin 10 mg; folic acid 2.5 mg; choline chloride 1200 mg; Manganese 15 mg; Zinc 60 mg; Iron 38 mg; Copper 5 mg; Selenium 0.1 mg; Iodine 0.2 mg; Selenium 0.05 mg; (^b,c,e,f^): Calculated on the basis of the ingredients composition. (^d^) Digestible energy (DE) was calculated according to Lebas [21] using the following equation: DE = 15.627 + 0.000982 CP^2^ + 0.0040 EE^2^ − 0.0114 MM^2^ − 0.169 ADF ± 1.250 MJ/kg DM. DM = Dry matter; CP = %crude protein in DM; EE = % ether extract (lipids) in DM; MM =% minerals (ash) in DM; ADF = % acid detergent fibre in DM; CF = % crude fibre in DM.

**Table 2:** Effect of dietary Zn-Sp, Se-Sp and their combination on the growth performance of growing rabbits.

Items	Control	Supplementation (mg/kg DM)			SEM	*p*-Value
		Zn-Sp	Se-Sp	Zn-Se-Sp		
Average body weight, (g)						
at 6 week	745.73	745.33	745.60	746.00	31.27	1.000
at 10 week	1460.66	1515.66	1551.33	1499.33	30.79	0.22
at 14 week	2034.33 ^b^	2169.66 ^a^	2199.33 ^a^	2200.33 ^a^	30.13	0.005
Average daily weight gain (g/rabbit/day)						
6–10 week	25.53 ^b^	27.51 ^ab^	28.77 ^a^	26.90 ^ab^	0.81	0.05
10–14 week	20.48 ^b^	23.35 ^ab^	23.14 ^ab^	25.03 ^a^	1.06	0.03
6–14 week	23.01 ^b^	25.43 ^a^	25.96 ^a^	25.97 ^a^	0.66	0.006
Average daily feed intake (g/rabbit/day)						
6–8 week	99.04	97.65	96.16	98.78	4.59	0.96
10–14 week	99.98 ^a^	96.33 ^ab^	86.98 ^c^	91.21 ^bc^	2.41	0.002
6–14 week	98.78	96.99	91.57	94.99	2.89	0.34
Feed conversion (g feed/g gain)						
6–8 week	3.87	3.55	3.34	3.67	0.19	0.24
10–14 week	4.88 ^a^	4.12 ^b^	3.75 ^b^	3.64 ^b^	0.22	0.0005
6–14 week	4.29 ^a^	3.81 ^b^	3.52 ^b^	3.65 ^b^	0.14	0.002

^a,b,c^ Mean values with the same letter within the same row did not differ significantly (*p* > 0.05). SEM: Standard Error of Means.

**Table 3:** Effect of dietary supplemental Zn-Sp, Se-algae and their combination on digestibility and nutritive value of experimental rabbit diets.

Supplementation (mg/kg DM)							
Item	Control	Zn-Sp	Se-Sp	Zn-Se-Sp		±SEM	*p*-Value
**Digestibility (%)**							
DM	62.38 ^b^	73.23 ^a^	67.77 ^ab^		70.86 ^ab^	2.65	0.09
OM	64.26 ^b^	74.75 ^a^	69.71 ^ab^		72.71 ^ab^	2.55	0.08
CP	66.23 ^b^	75.25 ^a^	70.94 ^ab^		73.83 ^a^	2.51	0.13
CF	49.25	62.05	54.07		55.27	4.72	0.35
EE	71.42 ^b^	77.92 ^a^	73.28 ^ab^		78.02 ^a^	1.53	0.03
NFE	67.41 ^b^	78.11 ^a^	73.66 ^ab^		76.96 ^a^	2.60	0.07
**Nutritive Values**							
DCP (g)	11.78	13.02	12.28		12.77	0.46	0.23
TDN (%)	64.61	72.93	67.56		71.06	2.88	0.31
DE(kcal/kg)	2862.22	3230.79	2992.91		3147.95	127.75	0.25

^a,b^ Means in the same row with different superscripts are significantly different (*p* < 0.05). SEM: Standard Error of Means.

**Table 4:** Effect of different experimental diets on carcass traits and meat chemical composition of growing rabbits.

Supplementation (mg/kg DM)						
Items	Control	Zn-Sp	Se-Sp	Zn-Se-Sp	±SEM	*p*-Value
Carcass traits						
Pre-slaughter weight (g)	2000.0	2078.33	2046.67	2181.00	27.59	0.21
Hot carcass weight (g)	1070.56 ^c^	1251.93 ^b^	1229.67 ^b^	1366.43 ^a^	32.67	0.001
Dressing %	53.52 ^b^	60.27 ^a^	60.13 ^ab^	65.68 ^a^	1.73	0.01
Liver weight %	3.19 ^a^	2.73 ^ab^	2.46 ^b^	2.29 ^b^	0.19	0.05
Heart weight %	0.38 ^a^	0.36 ^ab^	0.37 ^a^	0.31 ^b^	0.01	0.06
Kidney weight %	0.64 ^ab^	0.67 ^a^	0.59 ^ab^	0.56 ^b^	0.02	0.01
Spleen%	0.11	0.12	0.12	0.18	0.02	0.25
Edible Giblets ^1^ %	4.21 ^a^	3.76 ^ab^	3.45 ^ab^	3.17 ^b^	0.22	0.05
Total edible parts ^2^ %	57.73 ^b^	64.03 ^ab^	63.59 ^ab^	68.85 ^a^	1.86	0.01
Non- edible parts %	42.27 ^a^	35.97 ^ab^	39.38 ^ab^	34.30 ^b^	1.77	0.01
Meat chemical composition (%)						
DM	27.87 ^a^	27.32 ^ab^	26.85 ^b^	26.91 ^ab^	0.26	0.07
CP	22.12	23.14	22.80	22.84	0.31	0.19
EE	3.67 ^a^	2.70 ^b^	2.49 ^bc^	2.65 ^c^	0.24	0.02
Ash	2.09	1.48	1.56	1.42	0.22	0.19
Zn and Se content in meat						
Zn (mg/100 g)	0.95 ^b^	1.57 ^a^	0.96 ^b^	1.58 ^a^	0.009	0.0001
Se (µg/g)	0.094 ^b^	0.097 ^b^	0.46 ^a^	0.47 ^a^	0.003	0.0001

^a,b,c^ Mean values with the same letter within the same row did not differ significantly (*p* > 0.05); ^1^ Edible Giblets, % = (Liver+ kidneys + heart)/ Pre-slaughter weight (g)×100; ^2^ Total edible parts, % = (Carcass weight + edible giblets weight)/Pre-slaughter weight (g) × 100.

**Table 5:** Effect of dietary supplemental Zn-Sp, Se-Sp and their combination on plasma biochemicals and antioxidative status of the experimental growing rabbits.

Items	Supplementation (mg/kg DM)				±SEM	*p*-Value
	control	Zn-Sp	Se-Sp	Zn-Se-Sp		
Plasma biochemicals						
Total protein (g/dL)	6.20 ^b^	6.68 ^ab^	6.90 ^ab^	7.33 ^a^	0.22	0.02
Albumin (g/dL)	3.72	3.93	3.25	3.65	0.25	0.31
Globulin (g/dL)	2.47 ^b^	2.75 ^ab^	3.65 ^a^	3.68 ^a^	0.31	0.03
Albumin / Globulin ratio	1.51	1.43	0.89	0.99	0.20	0.10
AST	33.37	34.81	37.20	35.07	2.05	0.63
ALT	44.00	44.50	43.37	46.12	1.51	0.62
Total lipids (mg/dL)	292.24 ^a^	244.60 ^b^	225.80 ^b^	232.30 ^b^	7.04	0.0001
Triglycerides (mg/dL)	73.44 ^a^	67.85 ^ab^	60.44 ^b^	59.15 ^b^	2.81	0.01
Total cholesterol (mg/dL)	107.74 ^a^	94.17 ^ab^	86.40 ^b^	80.36 ^b^	6.39	0.05
HDL (mg/dL)	24.42 ^c^	27.03 ^bc^	28.44 ^b^	32.53 ^a^	1.13	0.002
LDL (mg/dL)	68.63 ^a^	53.57 ^ab^	45.87 ^b^	36.00 ^b^	6.33	0.02
VLDL (mg/dL)	14.68 ^a^	13.57 ^ab^	12.09 ^b^	11.83 ^b^	0.56	0.011
Antioxidative status						
T-AOC (mmol/L)	0.59 ^c^	0.95 ^bc^	1.07 ^b^	1.97 ^a^	0.14	0.0001
TBARs	6.47 ^a^	6.08 ^a^	5.17 ^b^	2.95 ^c^	0.15	0.0001
GSH-Px (U/L)	0.92 ^b^	1.07 ^b^	1.20 ^b^	2.00 ^a^	0.16	0.0020
Catlase (U/L)	495.07 ^b^	571.78 ^ab^	635.73 ^a^	623.48 ^a^	30.91	0.0285
SOD (U/L)	28.98 ^c^	33.29 ^bc^	40.43 ^ab^	45.57 ^a^	2.68	0.0075

^a,b,c^ Mean values with the same letter within the same row did not differ significantly (*p* > 0.05). Total antioxidant capacity (mmol/L), TBARS, thiobarbituric acid reactive substances, Glutathione peroxidase (U/L) and Superoxide dismutase (U/L).

## Data Availability

The data that support the findings of this study are available on request from the corresponding author. The data are not publicly available due to privacy or ethical restrictions.
